# Crystal structure of 4,4′-di­bromo-2′,5′-dimeth­oxy-[1,1′-biphen­yl]-2,5-dione (BrHBQBr)

**DOI:** 10.1107/S2056989015020472

**Published:** 2015-11-04

**Authors:** Joseph E. Meany, Steven P. Kelley, Robert M. Metzger, Robin D. Rogers, Stephen A. Woski

**Affiliations:** a Department of Chemistry, The University of Alabama, Box 870336, Tuscaloosa, AL 35487-0336, USA; b Department of Chemistry, McGill University, 801 Sherbrooke St. West, Montreal, Quebec, Canada, H3A 0B8

**Keywords:** crystal structure, mol­ecular rectifier, hemibi­quinone, π–π stacking inter­actions, hydrogen bonding, Br⋯Br halogen bonding

## Abstract

In the title compound, C_14_H_10_Br_2_O_4_, the dihedral angle between the aromatic rings is 67.29 (19)°. Both meth­oxy-group C atoms lie close to the plane of their attached ring [deviations = −0.130 (4) and 0.005 (5) Å]. In the crystal, mol­ecules pack in a centrosymmetric fashion and inter­act *via* a mixture of weak π–π stacking inter­actions [centroid–centoid separations = 4.044 (2) and 4.063 (3) Å], weak C—H⋯O hydrogen bonding, and Br⋯Br halogen bonding. This induces a geometry quite different than that predicted by theory.

## Abstract 2

In the crystal, mol­ecules pack in a centrosymmetric fashion and inter­act *via* a mixture of weak π–π stacking inter­actions, weak C—H⋯O hydrogen bonding, and Br⋯Br halogen bonding. This induces a geometry quite different than that predicted by theory.

## Chemical context   

Biphenyl derivatives have recently been investigated as conductors for single mol­ecule electronic systems (Venkataraman *et al.*, 2006 ▸). Researchers have shown that as the equilibrium twist angle θ between the two rings increases, conduction through the mol­ecule decreases as cos ^2^(θ). This effect is rationalized as a loss of overlap between two π systems. Inter­rupting conjugation is a prerequisite for the design of unimolecular rectifiers (Aviram & Ratner, 1974 ▸). Biphenyl derivatives with one electron-rich and one electron-deficient ring may be able to bias the direction of electron flow through the mol­ecule, thus acting as a mol­ecular diode. To this end we propose a di­meth­oxy­benzene-quinone structure (‘hemibi­quinone’, HBQ) as a potential unimolecular device. The asymmetric biphenyl structure should allow for high conductivity through each of the rings, while the dihedral angle between the two rings decreases orbital overlap and allows for partial isolation of the electron-rich donor and electron-poor acceptor moieties.
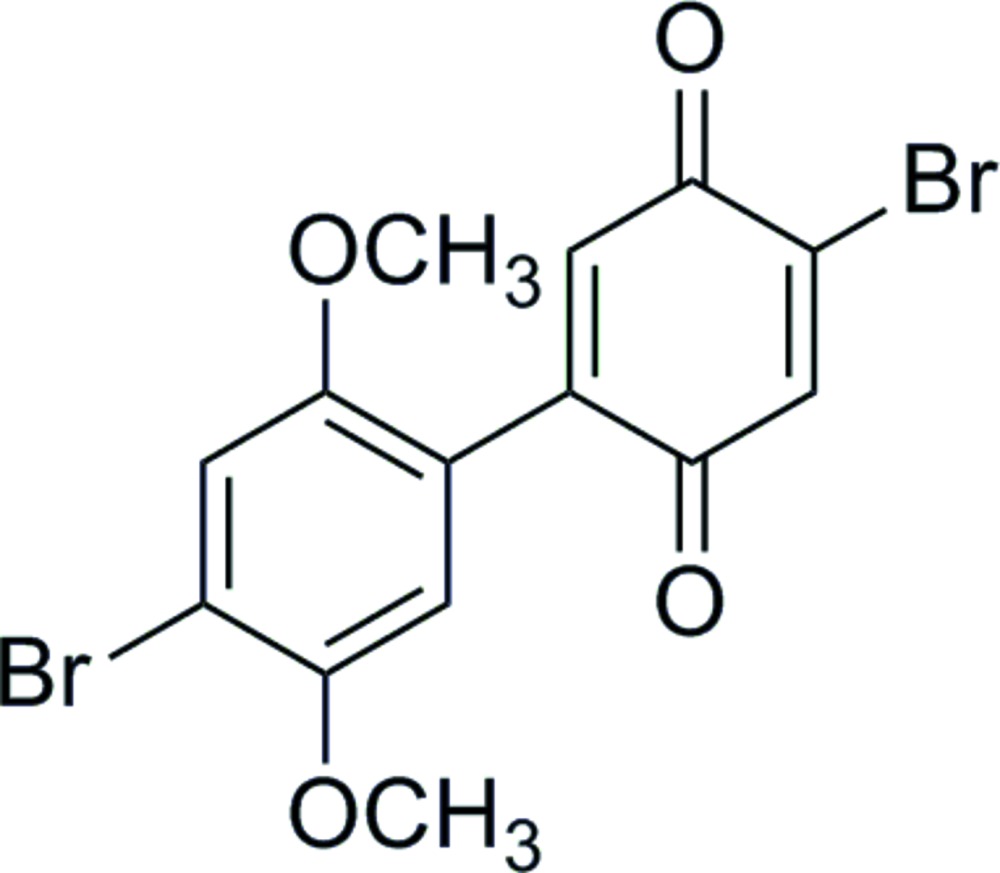


**Figure 1:**
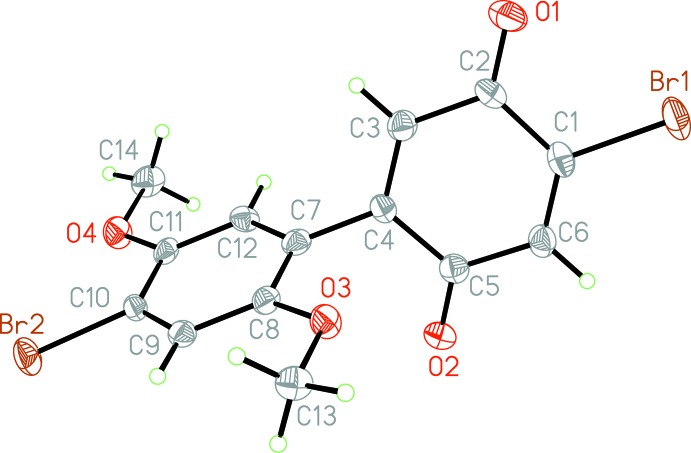
The mol­ecular structure of the title compound, showing displacement ellipsoids at the 50% probability level.

Two HBQ structures have been previously reported by Taylor *et al.* (2007 ▸) and Zeng & Becker (2004 ▸). The mol­ecule described herein is unique in that it possesses bromine substituents on each ring (Fig. 1 ▸). The distal halogens allow for high synthetic versatility: these groups can be elaborated sequentially with functional groups to allow deposition in a predictable manner onto a variety of substrates. Originally this mol­ecule was proposed by Love *et al.* (2009 ▸) as an impurity in the synthesis of 4,4′-di­bromo­diquinone; however, characterization of this compound was not reported. We have developed a selective synthesis for this hemibi­quinone that is scalable to gram qu­anti­ties.

## Structural commentary   

Because of the crucial role that the twist angle between the rings plays in the electronic properties of the mol­ecule, the determination of the C12—C7—C4—C5 torsion angle is the key observation in this structural analysis. This angle measures −110.9 (4)° in the crystal structure. DFT (B3LYP-DGDZVP) calculations performed on the target mol­ecule in the gas phase predict an angle of −38.54°. This significant discrepancy is probably due to packing inter­actions in the solid phase.

Substituents on the HBQ system behave as expected. The C—Br bond distances reflect the natures of the electron-deficient quinone and electron-rich di­meth­oxy­benzene rings: the C1—Br1 bond distance is 1.872 (5) Å, while the C10—Br2 bond is 1.897 (4) Å. Thus Br1 has a slightly stronger π-donating character into the quinone moiety, strengthening the bond relative to the C10—Br2 bond of the di­meth­oxy­benzene ring. The meth­oxy substituents are nearly coplanar to the benzene ring, with a C12—C11—O4—C14 torsion angle of 1.5 (6)° and a C9—C8—O3—C13 torsion angle of −4.4 (5)°. The methyl portions of each of these groups point away from the sterically restricting groups *ortho* to these positions. Finally, the quinone ring is slightly buckled (r.m.s. deviation = 0.064 Å), probably due to supra­molecular packing effects.

## Supra­molecular features   

**Figure 2:**
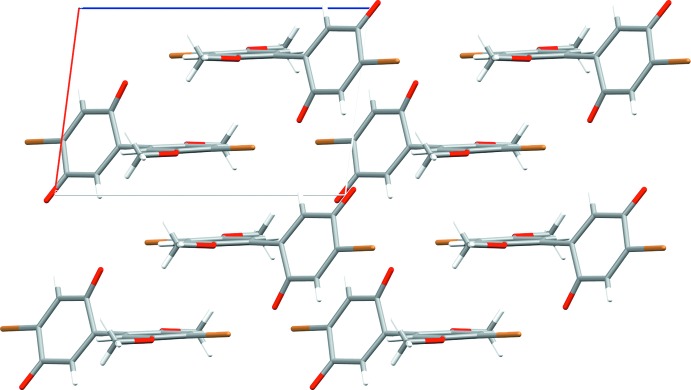
The unit-cell packing of the title compound, viewed down the *b* axis.

Each mol­ecule is surrounded by eight neighboring mol­ecules, which inter­act through hydrogen bonding, halogen bonding, and π–π inter­actions (Figs. 2 ▸ and 3 ▸). The strongest inter­actions appear to be between functional groups on the quinone ring of one mol­ecule with those on the di­meth­oxy­benzene ring of another. These include especially short but non-directional C—H⋯O hydrogen bonds (Table 1 ▸) between the quinone carbonyl groups and di­meth­oxy­benzene ring hydrogen atoms of two neighbors. Inter­actions between like parts of neighboring mol­ecules include edge-to-edge stacking of quinone rings with quinone rings, di­meth­oxy­benzene rings with di­meth­oxy­benzene rings, and dimeric hydrogen bonding between meth­oxy groups. Quinone rings on adjacent mol­ecules along the *c* axis show some face-to-face π-stacking.

Along the *a* axis, the benzene rings ‘nestle’ closely to one another in an anti­parallel geometry, where one quinone points up and the layer behind it points down. Within the *cb* plane, the benzene rings are coplanar; hydrogen atoms from C14 on one mol­ecule project closely to O3 on the adjacent mol­ecule and *vice versa* for a hydrogen atom attached to C13 to the adjacent O4 (Fig. 2 ▸). Symmetric C—H⋯π short contacts exist between pairs of C13—H13*C*⋯di­meth­oxy­benzene (Table 1 ▸).

**Figure 3:**
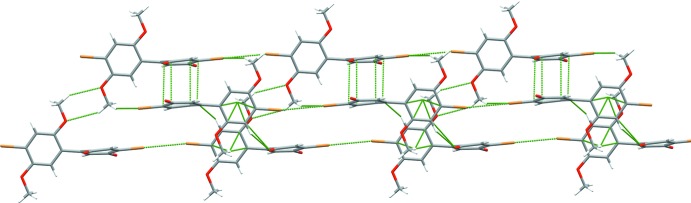
Packing diagram showing the stacking of parallel halogen-bonded chains. The view is down the *a* axis.

Mol­ecules are aligned linearly in a head-to-tail manner where the bromine atoms participate in Br⋯Br halogen bonding (Fig. 3 ▸). As discussed above, Br1 is electron deficient with respect to Br2, and a distinct halogen bond forms along the mol­ecular *x-*axis (the C7—C4 biphenyl bond). The Br1⋯Br2 separation is 3.4204 (8) Å, with almost linear C1—Br1⋯Br2 and C10—Br2⋯Br1 angles of 178.2 (4) and 170.9 (4)°, respectively. Equivalent rings from mol­ecules packed along this axis are parallel to one another; the quinone and benzene rings aligned coplanar to the corresponding ring in the next mol­ecule.

## Synthesis and crystallization   

Cerium(IV) ammonium nitrate (0.956 g, 1.75 mmol, 1.75 eq) was dissolved in 30 ml of H_2_O. A solution of 2-bromo-1,4-di­meth­oxy­benzene (0.253 g, 1.17 mmol) in 25 ml of aceto­nitrile was quickly added with vigorous stirring. After three hours, the product had precipitated as a grey–green powder. The precipitate was filtered, washed with water, and dried. The crude product was purified using flash chromatography (silica gel, chloro­form), yielding 0.0959 g of the desired product (20.3%). Crystals were obtained by slow evaporation of a solution in chloro­form.

## Refinement   

Hydrogen atoms were placed in calculated positions, and their coordinates and displacement parameters were constrained to ride on the carrier atom [C—H = 0.98 Å and *U*_iso_(H) = 1.5*U*_eq_(C) for methyl H atoms, C—H = 0.95 Å and *U*_iso_(H) = 1.5*U*_eq_(C) for other H atoms]. Hydrogen atoms on methyl groups were refined with a riding rotating model. Crystal data, data collection and structure refinement details are summarized in Table 2 ▸.

## Supplementary Material

Crystal structure: contains datablock(s) I. DOI: 10.1107/S2056989015020472/hb7514sup1.cif

Structure factors: contains datablock(s) I. DOI: 10.1107/S2056989015020472/hb7514Isup2.hkl

Supporting information file. DOI: 10.1107/S2056989015020472/hb7514Isup3.cml

CCDC reference: 1433845

Additional supporting information:  crystallographic information; 3D view; checkCIF report

## Figures and Tables

**Table 1:** Hydrogen-bond geometry (Å, °) *Cg*1 and *Cg*2 are the centroids of the C1–C6 and C7–C12 rings, respectively.

*D*—H⋯*A*	*D*—H	H⋯*A*	*D*⋯*A*	*D*—H⋯*A*
C9—H9*A*⋯O2^i^	0.95	2.40	3.270 (5)	151
C12—H12*A*⋯O1^ii^	0.95	2.62	3.461 (5)	148
C13—H13*A*⋯O4^iii^	0.98	2.65	3.525 (5)	149
C13—H13*B*⋯*Cg*2^i^	0.98	2.57	3.443 (5)	148
C14—H14*A*⋯*Cg*1^iv^	0.98	3.00	3.695 (5)	129

Symmetry codes: (i) 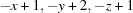; (ii) 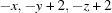; (iii) 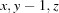; (iv) 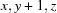.

**Table 2:** Experimental details

Crystal data	
Chemical formula	C_14_H_10_Br_2_O_4_
*M*_r_	402.04
Crystal system, space group	Triclinic, *P* 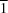
Temperature (K)	173
*a*, *b*, *c* (Å)	7.0909 (6), 9.2120 (8), 10.7056 (10)
α, β, γ (°)	90.989 (3), 97.098 (3), 101.909 (3)
*V* (Å^3^)	678.35 (10)
*Z*	2
Radiation type	Mo *K*α
μ (mm^−1^)	5.98
Crystal size (mm)	0.10 × 0.07 × 0.06

Data collection	
Diffractometer	Bruker APEXII CCD
Absorption correction	Multi-scan (*AXScale*; Bruker, 2010 ▸)
*T*_min_, *T*_max_	0.561, 0.745
No. of measured, independent and observed [*I* > 2σ(*I*)] reflections	7453, 2714, 2180
*R*_int_	0.036
(sin θ/λ)_max_ (Å^−1^)	0.630

Refinement	
*R*[*F* ^2^ > 2σ(*F* ^2^)], *wR*(*F* ^2^), *S*	0.043, 0.148, 1.04
No. of reflections	2714
No. of parameters	183
H-atom treatment	H-atom parameters constrained
Δρ_max_, Δρ_min_ (e Å^−3^)	0.67, −1.06

Computer programs: *APEX2* and *SAINT* (Bruker, 2010 ▸), *SHELXS97* and *SHELXTL* (Sheldrick, 2008 ▸) and *SHELXL2014* (Sheldrick, 2015 ▸).

## supplementary crystallographic information

### Crystal data

**Table:**

C_14_H_10_Br_2_O_4_	*Z* = 2
*M**_r_* = 402.04	*F*(000) = 392
Triclinic, *P*1	*D*_x_ = 1.968 Mg m^−^^3^
*a* = 7.0909 (6) Å	Mo *K*α radiation, λ = 0.71073 Å
*b* = 9.2120 (8) Å	Cell parameters from 3169 reflections
*c* = 10.7056 (10) Å	θ = 3.0–26.5°
α = 90.989 (3)°	µ = 5.98 mm^−^^1^
β = 97.098 (3)°	*T* = 173 K
γ = 101.909 (3)°	Fragment, brown
*V* = 678.35 (10) Å^3^	0.10 × 0.07 × 0.06 mm

### Data collection

**Table:**

Bruker APEXII CCD diffractometer	2180 reflections with *I* > 2σ(*I*)
φ and ω scans	*R*_int_ = 0.036
Absorption correction: multi-scan (*AXScale*; Bruker, 2010)	θ_max_ = 26.6°, θ_min_ = 1.9°
*T*_min_ = 0.561, *T*_max_ = 0.745	*h* = −8→8
7453 measured reflections	*k* = −11→11
2714 independent reflections	*l* = −13→13

### Refinement

**Table:**

Refinement on *F*^2^	Primary atom site location: structure-invariant direct methods
Least-squares matrix: full	Secondary atom site location: difference Fourier map
*R*[*F*^2^ > 2σ(*F*^2^)] = 0.043	Hydrogen site location: inferred from neighbouring sites
*wR*(*F*^2^) = 0.148	H-atom parameters constrained
*S* = 1.04	*w* = 1/[σ^2^(*F*_o_^2^) + (0.1053*P*)^2^] where *P* = (*F*_o_^2^ + 2*F*_c_^2^)/3
2714 reflections	(Δ/σ)_max_ = 0.001
183 parameters	Δρ_max_ = 0.67 e Å^−^^3^
0 restraints	Δρ_min_ = −1.06 e Å^−^^3^

### Special details

**Table:**

Geometry. All e.s.d.'s (except the e.s.d. in the dihedral angle between two l.s. planes) are estimated using the full covariance matrix. The cell e.s.d.'s are taken into account individually in the estimation of e.s.d.'s in distances, angles and torsion angles; correlations between e.s.d.'s in cell parameters are only used when they are defined by crystal symmetry. An approximate (isotropic) treatment of cell e.s.d.'s is used for estimating e.s.d.'s involving l.s. planes.

### Fractional atomic coordinates and isotropic or equivalent isotropic displacement parameters (Å^2^)

**Table:**

	*x*	*y*	*z*	*U*_iso_*/*U*_eq_	
Br1	0.28348 (7)	0.59976 (6)	1.11818 (5)	0.0442 (2)	
Br2	0.25434 (6)	1.33646 (5)	0.34039 (4)	0.0308 (2)	
O1	−0.0292 (5)	0.7668 (4)	1.0260 (3)	0.0365 (8)	
O2	0.5998 (4)	0.9672 (4)	0.8081 (3)	0.0282 (7)	
O3	0.2720 (4)	0.8360 (3)	0.5689 (3)	0.0246 (6)	
O4	0.2214 (4)	1.4265 (3)	0.6021 (3)	0.0272 (7)	
C1	0.2910 (6)	0.7438 (5)	0.9966 (4)	0.0258 (9)	
C2	0.1101 (6)	0.8048 (5)	0.9676 (4)	0.0239 (9)	
C3	0.1079 (6)	0.9086 (5)	0.8659 (4)	0.0240 (9)	
H3A	−0.0054	0.9476	0.8449	0.029*	
C4	0.2593 (6)	0.9515 (4)	0.8004 (4)	0.0189 (8)	
C5	0.4475 (6)	0.9059 (5)	0.8446 (4)	0.0223 (9)	
C6	0.4464 (6)	0.7913 (5)	0.9381 (4)	0.0243 (9)	
H6A	0.5583	0.7501	0.9572	0.029*	
C7	0.2536 (5)	1.0448 (5)	0.6908 (4)	0.0215 (9)	
C8	0.2612 (5)	0.9822 (4)	0.5715 (4)	0.0196 (8)	
C9	0.2575 (5)	1.0693 (4)	0.4676 (4)	0.0196 (8)	
H9A	0.2624	1.0282	0.3864	0.024*	
C10	0.2465 (5)	1.2161 (5)	0.4825 (4)	0.0200 (8)	
C11	0.2352 (5)	1.2807 (4)	0.5986 (4)	0.0193 (8)	
C12	0.2373 (5)	1.1929 (5)	0.7029 (4)	0.0207 (8)	
H12A	0.2275	1.2340	0.7833	0.025*	
C13	0.2659 (6)	0.7645 (5)	0.4477 (4)	0.0241 (9)	
H13A	0.2659	0.6590	0.4580	0.036*	
H13B	0.3801	0.8112	0.4087	0.036*	
H13C	0.1476	0.7745	0.3938	0.036*	
C14	0.2129 (7)	1.4946 (5)	0.7220 (5)	0.0305 (10)	
H14A	0.1988	1.5974	0.7109	0.046*	
H14B	0.3327	1.4938	0.7781	0.046*	
H14C	0.1013	1.4394	0.7590	0.046*	

### Atomic displacement parameters (Å^2^)

**Table:**

	*U*^11^	*U*^22^	*U*^33^	*U*^12^	*U*^13^	*U*^23^
Br1	0.0440 (3)	0.0453 (4)	0.0449 (4)	0.0096 (2)	0.0080 (2)	0.0305 (3)
Br2	0.0436 (3)	0.0247 (3)	0.0258 (3)	0.0094 (2)	0.0065 (2)	0.01177 (19)
O1	0.0362 (17)	0.037 (2)	0.041 (2)	0.0078 (14)	0.0193 (15)	0.0106 (15)
O2	0.0248 (14)	0.0329 (18)	0.0288 (18)	0.0059 (12)	0.0101 (12)	0.0073 (13)
O3	0.0345 (16)	0.0171 (15)	0.0247 (17)	0.0089 (12)	0.0075 (12)	0.0024 (12)
O4	0.0369 (16)	0.0190 (16)	0.0287 (18)	0.0109 (12)	0.0071 (13)	0.0038 (13)
C1	0.031 (2)	0.020 (2)	0.025 (2)	0.0029 (17)	0.0031 (17)	0.0069 (18)
C2	0.027 (2)	0.023 (2)	0.022 (2)	0.0014 (16)	0.0082 (16)	0.0036 (17)
C3	0.0241 (19)	0.019 (2)	0.029 (3)	0.0056 (16)	0.0047 (17)	0.0031 (17)
C4	0.0259 (19)	0.016 (2)	0.016 (2)	0.0052 (15)	0.0046 (15)	0.0002 (15)
C5	0.026 (2)	0.021 (2)	0.020 (2)	0.0059 (16)	0.0037 (16)	−0.0010 (16)
C6	0.028 (2)	0.026 (2)	0.019 (2)	0.0078 (17)	0.0007 (16)	0.0027 (17)
C7	0.0158 (17)	0.019 (2)	0.030 (2)	0.0029 (14)	0.0052 (15)	0.0037 (17)
C8	0.0177 (17)	0.015 (2)	0.026 (2)	0.0034 (14)	0.0042 (15)	0.0040 (16)
C9	0.0176 (17)	0.019 (2)	0.023 (2)	0.0038 (14)	0.0047 (15)	0.0035 (16)
C10	0.0157 (17)	0.025 (2)	0.019 (2)	0.0036 (15)	0.0024 (14)	0.0079 (17)
C11	0.0169 (17)	0.0135 (19)	0.028 (2)	0.0048 (14)	0.0035 (15)	0.0052 (16)
C12	0.0210 (18)	0.016 (2)	0.025 (2)	0.0017 (14)	0.0068 (15)	0.0011 (16)
C13	0.026 (2)	0.019 (2)	0.027 (2)	0.0031 (16)	0.0047 (16)	−0.0037 (17)
C14	0.035 (2)	0.020 (2)	0.039 (3)	0.0101 (18)	0.0077 (19)	−0.003 (2)

### Geometric parameters (Å, º)

**Table:**

Br1—C1	1.872 (5)	C6—H6A	0.9500
Br2—C10	1.897 (4)	C7—C12	1.398 (6)
O1—C2	1.227 (5)	C7—C8	1.404 (6)
O2—C5	1.224 (5)	C8—C9	1.384 (6)
O3—C8	1.365 (5)	C9—C10	1.378 (6)
O3—C13	1.437 (5)	C9—H9A	0.9500
O4—C11	1.367 (5)	C10—C11	1.388 (6)
O4—C14	1.433 (6)	C11—C12	1.390 (6)
C1—C6	1.334 (6)	C12—H12A	0.9500
C1—C2	1.504 (6)	C13—H13A	0.9800
C2—C3	1.462 (6)	C13—H13B	0.9800
C3—C4	1.349 (6)	C13—H13C	0.9800
C3—H3A	0.9500	C14—H14A	0.9800
C4—C7	1.468 (6)	C14—H14B	0.9800
C4—C5	1.504 (6)	C14—H14C	0.9800
C5—C6	1.467 (6)		
			
C8—O3—C13	117.3 (3)	C9—C8—C7	119.4 (4)
C11—O4—C14	117.8 (3)	C10—C9—C8	119.7 (4)
C6—C1—C2	121.0 (4)	C10—C9—H9A	120.2
C6—C1—Br1	122.8 (4)	C8—C9—H9A	120.2
C2—C1—Br1	116.2 (3)	C9—C10—C11	122.4 (4)
O1—C2—C3	122.1 (4)	C9—C10—Br2	119.1 (3)
O1—C2—C1	121.0 (4)	C11—C10—Br2	118.5 (3)
C3—C2—C1	116.8 (4)	O4—C11—C10	117.4 (4)
C4—C3—C2	123.1 (4)	O4—C11—C12	124.6 (4)
C4—C3—H3A	118.5	C10—C11—C12	118.0 (4)
C2—C3—H3A	118.5	C11—C12—C7	120.7 (4)
C3—C4—C7	123.8 (4)	C11—C12—H12A	119.7
C3—C4—C5	118.4 (4)	C7—C12—H12A	119.7
C7—C4—C5	117.8 (3)	O3—C13—H13A	109.5
O2—C5—C6	120.5 (4)	O3—C13—H13B	109.5
O2—C5—C4	121.1 (4)	H13A—C13—H13B	109.5
C6—C5—C4	118.3 (4)	O3—C13—H13C	109.5
C1—C6—C5	121.2 (4)	H13A—C13—H13C	109.5
C1—C6—H6A	119.4	H13B—C13—H13C	109.5
C5—C6—H6A	119.4	O4—C14—H14A	109.5
C12—C7—C8	119.8 (4)	O4—C14—H14B	109.5
C12—C7—C4	121.3 (4)	H14A—C14—H14B	109.5
C8—C7—C4	118.9 (3)	O4—C14—H14C	109.5
O3—C8—C9	125.2 (4)	H14A—C14—H14C	109.5
O3—C8—C7	115.4 (4)	H14B—C14—H14C	109.5

### Hydrogen-bond geometry (Å, º)

Cg1 and Cg2 are the centroids of the C1–C6 and C7–C12 rings, respectively.

**Table:**

*D*—H···*A*	*D*—H	H···*A*	*D*···*A*	*D*—H···*A*
C9—H9*A*···O2^i^	0.95	2.40	3.270 (5)	151
C12—H12*A*···O1^ii^	0.95	2.62	3.461 (5)	148
C13—H13*A*···O4^iii^	0.98	2.65	3.525 (5)	149
C13—H13*B*···*Cg*2^i^	0.98	2.57	3.443 (5)	148
C14—H14*A*···*Cg*1^iv^	0.98	3.00	3.695 (5)	129

Symmetry codes: (i) −*x*+1, −*y*+2, −*z*+1; (ii) −*x*, −*y*+2, −*z*+2; (iii) *x*, *y*−1, *z*; (iv) *x*, *y*+1, *z*.
